# Predictive factors for inadequate bowel preparation using low-volume polyethylene glycol (PEG) plus ascorbic acid for an outpatient colonoscopy

**DOI:** 10.1038/s41598-019-56107-5

**Published:** 2019-12-23

**Authors:** Seung Yong Shin, Kyeong Seon Ga, In Young Kim, Yoo Mi Park, Da Hyun Jung, Jie-Hyun Kim, Young Hoon Youn, Hyojin Park, Jae Jun Park

**Affiliations:** 1 Department of Internal Medicine, Gangnam Severance Hospital, Yonsei University College of Medicine, Seoul, Korea; 2 Health Promotion Center, Gangnam Severance Hospital, Yonsei University College of Medicine, Seoul, Korea; 3 Department of Internal Medicine, Chung-Ang University College of Medicine, Seoul, Korea

**Keywords:** Colonoscopy, Colon

## Abstract

Low-volume polyethylene glycol (PEG) plus ascorbic acid solutions are widely used for bowel cleansing before colonoscopy. This study aimed to investigate the pre-endoscopic predictive factors for inadequate preparation in subjects receiving low-volume PEG plus ascorbic acid. A prospective study was performed at Gangnam Severance Hospital, Korea, from June 2016 to December 2016. All participants received low-volume PEG plus ascorbic acid solutions for outpatient colonoscopy. The split-dose bowel preparation was administered in subject with morning colonoscopy while same day bowel preparation was used for afternoon colonoscopy. 715 patients were enrolled (mean age 56.1 years, 54.4% male), of which 138 (19.3%) had an inadequate bowel preparation. In multivariable analysis, cirrhosis (OR 4.943, 95% CI 1.191–20.515), low (less than 70%) compliance for three-day low-residual diet (OR 2.165, 95% CI 1.333–3.515), brown liquid rectal effluent (compared with clear or semi-clear effluent) (OR 7.604, 95% CI, 1.760–32.857), and longer time interval (≥2 hours) between last defecation and colonoscopic examination (OR 1.841, 95% CI, 1.190–2.849) were found as an independent predictors for inadequate preparation. These predictive factors may be useful in guiding additional intervention to improve quality of bowel preparation.

## Introduction

Adequate preparation of the bowels is a crucial prerequisite for a complete and successful colonoscopy. Inadequate bowel preparation is associated with prolonged procedure time, incomplete procedure, and low neoplastic lesion detection rate^1,2^. Although high volume (4 L) polyethylene glycol (PEG)-based solutions have been used for bowel cleansing for a long time^3,4^, the large volume and unpleasant salty taste frequently lead to poor patient compliance^5,6^. Because of these PEG solution drawbacks, a combination of PEG with other osmotic or stimulant agents has been attempted to reduce the required PEG solution volume^7^.

In response to this problem, a low-volume PEG (2 L) plus ascorbic acid treatment has recently been developed to complement the drawbacks of the standard PEG solution volume. The excess ascorbic acid, which cannot be absorbed and functions as an osmotic laxative in the bowel lumen, can reduce the required volume of PEG solution from 4 L to 2 L. Furthermore, the addition of ascorbic acid improves the taste^8^. Several studies have shown superior patient compliance and similar efficacy compared with the standard PEG^8–10^ and this novel regimen is now widely used for bowel cleansing before colonoscopy.

Although low-volume PEG (2 L) plus ascorbic acid has shown good efficacy, some patients still show inadequate bowel preparation with this new regimen. Identifying the risk factors for inadequate bowel preparation and cleansing agent intake is imperative to take the appropriate measures to reduce the number of patients with inadequate preparation. Several studies have reported the predictors associated with inadequate bowel preparation, including older age, female sex, diabetes, constipation, history of abdominal or gynecologic surgery, and compliance with preparation instructions^11–13^. However, most of these parameters are identified based on the classic, large volume regimen, and the risk factors for inadequate preparation in low-volume PEG plus ascorbic acid are not well understood. Therefore, the aim of this study was to investigate the pre-endoscopic predictive factors for inadequate preparation in patients receiving low-volume PEG plus ascorbic acid treatment.

## Results

### Baseline patient characteristics

During the study period, 715 patients were enrolled from outpatient colonoscopy. Cecal intubation was confirmed in all patients. Table 1 shows the baseline patient characteristics. The patient population consisted of 54.4% men and 45.6% women, with a mean age 56.1 years. Colonoscopy was performed in the morning in 619 (86.6%) patients. Clinical indications for colonoscopy included 88.3% in screening or surveillance and 11.7% in symptom or abnormal findings such as abdominal pain, constipation, diarrhea, hematochezia, and positive fecal occult blood test. A total of 114 (15.9%) patients had a history of constipation, and among these patients, 31 (27.1%) used a laxative for constipation. More than half (412, 57.6%) of the patients experienced a prior colonoscopy. A total of 221 (30.9%) patients underwent abdominal and/or pelvic surgery and 54 (7.6%) patients had a history of colon resection. A total of 691 (96.6%) patients reported that they had consumed 100% of the prescribed cleansing agent while the remaining 24 (3.4%) patients reported consuming between 75% and 100% of the cleansing solution. Less than 70% adherence to the low fiber diet was observed in 179 patients (25%). The characteristics of last rectal effluent were ‘clear’ in 389 (54.4%) patients, ‘semi-clear’ in 313 (43.8%) patients, and ‘brown liquid’ in 13 (1.8%) patients, while no patients reported ‘brown solid’ rectal effluent. In sum, 138 (19.3%) patients showed inadequate bowel preparation.

**Table 1:** Baseline demographic and clinical characteristics of overall participants.

Risk factor	Participants (n = 715)
Age, yr, mean ± SD	56.14 ± 14
Male, n (%)	389 (54.4)
BMI, kg/m^2^, mean ± SD	23.4 ± 3.3
**Smoking, n (%)**	
Current smoking	117 (16.4)
Ex-smoking	133 (18.6)
No smoking	465 (65.0)
Constipation, n (%)	114 (15.9)
History of laxatives use, n (%) of constipation	31 (27.1)
Hypertension, n (%)	116 (16.2)
Diabetes mellitus, n (%)	58 (8.1)
Liver cirrhosis, n (%)	4 (0.6)
Chronic kidney disease, n (%)	6 (0.8)
**Medication**	
Calcium channel blocker	75 (10.5)
Beta blocker	17 (2.4)
Benzodiazepine	3 (0.4)
Oral iron	1(0.1)
Opioid or narcotics	0 (0)
Surgical history of abdomen and/or pelvis, n (%)	221 (30.9)
Surgical history of colon resection, n (%)	54 (7.6)
**Objective for colonoscopy**	
Screening or surveillance, n (%)	631 (88.3)
Symptom or abnormal findings, n (%)	84 (11.7)
Previous experience of colonoscopy, n (%)	412 (57.6)
Previous inadequate bowel preparation, n (%)*	35 (14.1)*
Previous history of diverticulosis, n (%)*	13 (5.2)*
History of polypectomy, n (%)	181 (25.3)
**Proportion of preparation consumed, n (%)**	
100%	691 (96.6)
>75%, <100%	24 (3.4)
**Low-residue diet**	
>70% adherence low fiber diet	536 (75)
≤70% adherence low fiber diet	179 (25)
**Bowel movement (times after purgative intake)**	
0–2	5 (0.7)
3–5	66 (9.2)
6–10	522 (73)
10	122 (17.1)
**Time interval between last defection and colonoscopy, n (%)**	
<2 hr	384 (53.7)
≥2 hr	331 (46.3)
Characteristics of last rectal effluent	
Clear	389 (54.4)
Semi-clear	313 (43.8)
Brown liquid	13 (1.8)
Brown solid	0 (0)
Morning colonoscopy, n (%)	619 (86.6)
**Bowel preparation, n (%)**	
Adequate	577 (80.7)
Inadequate	138 (19.3)

SD, standard deviation; BMI, Body mass index; NA, not available.

*This data is based on 249 patients whose previous colonoscopy finding have been confirmed.

### Comparison between patients with adequate and inadequate bowel preparation

In a comparison study based on bowel preparation quality, a history of cirrhosis, adherence to the low-residue diet, time interval between last defecation and colonoscopic examination, and the characteristics of last rectal effluent were significantly different between patients with adequate and inadequate bowel preparation. The proportion of patients with a history of cirrhosis was higher in the inadequate bowel preparation group than in the adequate bowel preparation group (2.9% vs. 0.7%, P = 0.027). Patients who adhered to the low fiber diet were more frequently identified in the adequate bowel preparation group than in the inadequate bowel preparation group (77.3% vs. 65.2%, P = 0.003). Patients who underwent colonoscopy within 2 hours after the last defecation were found more frequently in the adequate bowel preparation group than in the inadequate bowel preparation group (56.8% vs. 40.6%, P = 0.001). Moreover, patients who reported ‘brown liquid’ as the last rectal effluent were more frequently found in the inadequate bowel preparation group (5.8% vs. 0.9%, P < 0.001). On the other hand, patient demographic variables including age, sex, body mass index, colonoscopy indication, previous experience of colonoscopy, and history of inadequate bowel preparation or diverticulosis showed no significant difference between the two groups. The total number of bowel movements did not differ between the two groups. Additionally, medical co-morbidities other than cirrhosis, co-medication, and history of abdomen and/or pelvis surgery and colonic segmental resection showed no differences between the two groups (Table 2).

**Table 2:** Comparison of patients’ demographic and clinical characteristics between patients with adequate preparation and inadequate preparation.

Risk factor	Adequate preparation (n = 577)	Inadequate Preparation (n = 138)	P-value
Age, yr, mean ± SD	55.9 ± 14.4	56.9 ± 13.4	0.478
Male, n (%)	307 (53.2)	82 (59.4)	0.188
BMI, kg/m^2^, mean ± SD	23.2 ± 3.2	23.8 ± 3.5	0.066
Smoking, n (%)			0.829
Current smoking	94 (16.3)	23 (16.7)	
Ex-smoking	105 (18.2)	28 (20.3)	
No smoking	378 (65.5)	87 (63)	
Constipation, n (%)	85 (14.7)	29 (21)	0.070
Laxatives use, n (%)	26 (4.5)	8(5.8)	0.540
Hypertension, n (%)	89 (15.4)	27 (19.6)	0.236
Diabetes mellitus, n (%)	43 (7.5)	15 (10.9)	0.187
Cirrhosis, n (%)	4 (0.7)	4 (2.9)	0.027
Chronic kidney disease, n (%)	6 (1.0)	0 (0.0)	0.229
Calcium channel blocker	58 (10.1)	17 (12.3)	0.668
Beta blocker	14 (2.4)	3 (2.2)	0.511
Benzodiazepine	1 (0.2)	2 (1.4)	0.100
Previous inadequate bowel preparation, n (%)*	28 (14.4)*	7 (13.0)*	0.794*
Previous history of diverticulosis, n (%)*	8 (4.1)*	5 (9.8)*	0.132*
Surgical history of abdomen and/or pelvis, n (%)	180 (31.2)	41 (29.7)	0.734
Surgical history of colon resection, n (%)	42 (9.1)	12 (10.9)	0.562
Objective for colonoscopy			0.515
Screening or surveillance, n (%)	507 (87.9)	124 (89.9)	
Symptom or abnormal findings, n (%)	70 (12.1)	14 (10.1)	
Low-residue diet			0.003
>70% adherence to low fiber diet	446 (77.3)	90 (65.2)	
≤70% adherence to low fiber die	131 (22.7)	48 (34.8)	
Bowel movement (times after purgative intake)			0.386
0–2	4 (0.7)	1 (0.7)	
3–5	48 (8.4)	18 (13.0)	
6–10	424 (73.2)	98 (71.0)	
>10	101 (17.7)	21 (15.2)	
Time interval between last defection and colonoscopy, n (%)			0.001
<2 hr	328 (56.8)	56 (40.6%)	
≥2 hr	249 (43.2)	82 (59.4%)	
Color of last rectal effluent			<0.001
Clear	315 (54.6)	74 (53.6)	
Semi-clear	257 (44.5)	56 (40.6)	
Brown liquid	5 (0.9)	8 (5.8)	
Brown solid	0 (0.0)	0 (0.0)	
Morning colonoscopy, n (%)	503 (87.2)	116 (84.1)	0.335

SD, standard deviation; BMI, Body mass index; NA, not available.

*This data is based on 249 patients whose previous colonoscopy finding have been confirmed.

### Multivariate analysis for inadequate bowel preparation risk factors in patients using low-volume PEG plus ascorbic acid

Multivariate analysis showed that cirrhosis (OR 4.943, 95% CI 1.191–20.515, P = 0.028), ≤70% adherence to the low fiber diet (OR 2.111, 95% CI 1.375–3.242, P = 0.001), brown liquid rectal effluent (OR 5.659, 95% CI, 1.730–18.513, P = 0.004), and ≥2 hours from last defecation to colonoscopic examination (OR 1.970, 95% CI 1.334–2.910, P = 0.001) were independent factors associated with inadequate bowel preparation (Table 3).

**Table 3:** Multivariate analysis for risk factors of inadequate preparation in patients using low-volume PEG plus ascorbic acid.

Predictive factor	OR	95% CI	P-value
Body mass index	1.043	0.985–1.106	0.151
Cirrhosis	4.943	1.191–20.515	0.028
Constipation	1.539	0.943–2.512	0.151
≤70% adherence low fiber diet	2.111	1.375–3.242	0.001
Brown liquid rectal effluent	5.659	1.730–18.513	0.004
≥2hrs from last defecation to colonoscopy	1.970	1.334–2.910	0.001

## Discussion

Our study shows that cirrhosis, lack of adherence to a three-day low-residual diet, brown liquid rectal effluent, and ≥2 hours from last defecation to colonoscopic examination were independent predictors of inadequate preparation in patients using low-volume PEG (2 L) plus ascorbic acid treatment for bowel preparation in outpatient colonoscopy.

Cirrhosis has been reported as a predictor of inadequate bowel preparation in previous studies^11,14^. Thus, standard bowel preparation was shown to be ineffective in cirrhotic patients^15^. Regarding this finding, several factors, including autonomic dysfunction, small bowel bacterial overgrowth, are suggested as causes which result in impaired intestinal motility^16,17^. Our study showed that cirrhosis is an independent predictor of poor bowel preparation under low-volume PEG (2 L) plus ascorbic acid preparation.

The adherence to low-residue diet is regarded as an important factor for successful preparation in colonoscopy, and dietary restrictions for fiber or high residue diet are included in most bowel preparation guidelines^18–20^. Delegge *et al*. reported that patients who adhered to a low-residue diet before colonoscopy showed significantly better colon cleansing demonstrating a higher proportion of patients with good or excellent preparation^21^. Another study also indicated that a high-residue diet 2 days prior colonoscopy is an independent risk factor for inadequate bowel preparation using the 4 L PEG-based regimen^18^. In line with these observations, our study showed that less than 70% of adherence to low fiber and residue diet increased the risk of inadequate preparation by about 2-fold. To date, the PEG-based split regimen is known as the most potent regimen with a high degree of cleanliness. Our data indicate that the adherence to low fiber and low residue diet in the PEG-based split-dose regimen is still crucial for determining degree of bowel preparation.

Although patients were advised to follow the low fiber and residue diet for 3 days before examination and education leaflets for this preparation were distributed, 25% of patients showed less than 70% compliance to these instructions in our study. This result suggests that more efficient dietary education is needed in patients undergoing outpatient-based low-volume PEG ascorbic acid bowel preparation. As reported in previous studies, dietary adherence education using visual media in addition to verbal instruction and hand-out distribution may be helpful in improving the degree of bowel cleanliness^22,23^. Moreover, encouraging and reminding patients of dietary compliance through a short message service or by telephone may also be considered^24^.

Regarding the association between last rectal effluent characteristics and degree of bowel preparation, Fatima *et al*. showed that patients reporting their last effluent as brown liquid or solid have a substantial likelihood for inadequate preparation, comprising 54% of these patients having fair or poor preparation when they were given PEG or sodium phosphate solution with or without a split-dose regimen^25^. Nevertheless, the last rectal effluent variable was not analyzed under the control of several confounding variables in previous studies. In this study, the brown liquid last rectal effluent variable was found as an independent predictor for inadequate preparation (OR 5.659, 95% CI, 1.730–18.513, P = 0.004). This finding suggests that characteristic of the last rectal effluent is a robust predictor of poor preparation in outpatient colonoscopy. Considering this finding, features of the last rectal effluent before colonoscopy can be applied to screen subjects with a high probability of inadequate preparation, and additional colon cleansing using large-volume enemas or extra oral purgatives should be considered before examination^26^, however, the efficacy of these additional interventions needs to be confirmed by future studies.

In addition to last rectal effluent characteristic, time from last bowel movement to examination greater than 2 hours was confirmed as a risk factor for inadequate bowel preparation. This is a novel finding of our study. Here, the time interval between last purgative and colonoscopic examination was adjusted to within 5 hours in all study populations. However, the possibility of inadequate preparation increased when the time interval from last defecation to examination was prolonged. At present, it is unclear why this variable is related to inadequate preparation, but the influence of bile influx from the upper gastrointestinal tract on intervening time may be involved. Identifying the time of the last bowel movement before colonoscopy may also be helpful in stratifying patients with high risk of poor preparation.

According to a study of colonoscopy preparation in children, the total number of stool defecation was associated with the degree of cleanliness^27^. In a study by Safder *et al*., 149 children scheduled for colonoscopy were given PEG solution over a 4-day preparation period, and a simple questionnaire was completed by their parents. A total of ≥5 stools/day was one of the predictive factors associated with adequate preparation. However, in our study, the number of bowel movements after the taking oral purgatives was not related to preparation quality. Similar to our study, another study showed that the number of bowel movements during bowel cleansing was not related to degree of bowel preparation^28^. Although the reason for these discrepant results is unclear at present, prediction of bowel cleanliness based on the number of bowel movements may be useful only in children rather than adults.

In previous studies, old age, female sex, constipation, diabetes mellitus, stroke, co-medication, history of inadequate bowel preparation or diverticulosis, history of abdominal and/or pelvic surgery were shown as independent risk factors for inadequate bowel preparation^11,29–32^. However, these factors were not associated with degree of colonic cleanliness in our study. A possible explanation for these observations might be related to a recent meta-analysis that demonstrated that split-dosing was superior to non-split regimens for bowel preparation^33^. Most previous studies did not apply a split-dosing regimen and used the administration of various kinds of purgatives. However, in our study, the majority of patients underwent morning colonoscopy and were administrated the split-dosing regimen using low volume PEG and ascorbic acid. Strict control of the time interval between last purgative intake and colonoscopic examination in our study was also considered the cause of these differences. Several studies have shown that the time interval between last intake of purgative and colonoscopic examination is a crucial determinant for bowel cleansing, and the optimal time interval was reported as 3 to 5 hours for bowel preparation^34^. In our study, the time interval was restricted to within 5 hours in all study populations. This time interval was not considered as a factor affecting bowel cleansing, and was not a controlled factor in most of aforementioned previous studies. Thus, the optimal settings of our study, including split-dosing regimen and strict control of time interval, are thought to be the cause of reducing the effect of predictors found in previous studies.

There are several limitations to our study. First, this is a single-center study. Second, data availability may have biased some outcomes, as data regarding past medical and surgical histories and use of medications were retrospectively gathered by patient recall or medical record. For example, the results of patients who underwent colonoscopy at other hospitals more than 5 years ago were often difficult to assess or analyze properly. Third, since the evaluation of bowel preparation status was performed by five endoscopists, inter-observer variation could have occurred; however, we tried to minimize inter-observer variability for assessment of bowel cleanliness through repeated group meetings and practice assessments for all five endoscopists. Fourth, patient dietary assessment was rated as a 100-point scale; therefore, subjective intervention of each patient could exist. The amount of water that patients may have consumed in addition to the recommended amount was also not measured. Lastly, older patients may have difficulties in bowel preparation resulting in poor preparation. However, our study included relatively fewer older patients, and this may have affected the results. Nevertheless, to the best of our knowledge, it is the first study to evaluate the risk factors associated with inadequate bowel preparation in patients using low-volume PEG (2 L) plus ascorbic acid. Moreover, this study has the advantage of prospectively evaluating most of the variables associated with bowel cleansing in a relatively large number of patients.

In conclusion, cirrhosis, lack of adherence to the recommended low-residue diet, brown liquid rectal effluent, and longer interval between last defecation and examination are independent risk factors for inadequate bowel preparation using low-volume PEG (2 L) plus ascorbic acid for outpatient colonoscopy. These predictive factors may be useful for clinicians in developing additional interventions to improve the quality of bowel preparation.

## Materials and Methods

### Subjects

This study was conducted at a single center, Gangnam Severance Hospital, Korea, from June 2016 to December 2016. We prospectively enrolled consecutive outpatients between the ages of 18 and 80 years who were scheduled for colonoscopy with variable indications. Patients with the following clinical features were excluded: age younger than 18 years, pregnancy, breastfeeding, allergic to any preparation components, consuming less than 75% of the preparation solution, history of total colectomy, and refusal to participate in the study. All patients provided written informed consent. The study protocol conformed to the ethical guidelines of the 1975 Helsinki Declaration and was approved by the Institutional Review Board of Gangnam Severance hospital, Seoul, Korea (approval no. 3–2013–0134). The methods were carried out in accordance with the relevant guidelines and regulations, and the informed consent was obtained from all participants

### Bowel preparation

All patients were instructed to follow a low-residue diet for 3 days before the colonoscopy. Each patient received the list of dietary restrictions, including non-digestible and high fiber foods such as vegetables, fruits, grains. Patients were permitted a regular diet for breakfast and lunch but only an early soft dinner the day before the colonoscopy. Only clear liquids were allowed for the 2 hours before the procedure on the day of the colonoscopy. All participants received low-volume PEG (2 L) plus ascorbic acid solutions (Coolprep®; TaeJoon Pharmaceuticals, Seoul, Korea) for outpatient colonoscopy and were instructed how to prepare and consume the solution. Moreover, detailed instructions for the cleansing method was given to all participants. The split-dose bowel preparation was administered for patients undergoing morning colonoscopies while the conventional same-day preparation was used for afternoon colonoscopies. Colonoscopy starting before 12:00 p.m. was defined as morning colonoscopy and starting after 13:30 p.m. was defined as afternoon colonoscopy. We divided morning colonoscopy patients into two groups based on the procedure occurring before or after 10:30 a.m. and applied different methods of bowel preparation solution consumption to each group to restrict and limit the time interval between last intake of purgative and colonoscopic examination to within 5 hours. All patients with morning colonoscopy started ingesting the first half of the solution (1 L) at 21:00 p.m. the previous evening, but patients scheduled before 10:30 a.m. in the morning started the second half of the solution (1 L) at 05:00 a.m. the day of colonoscopy, and patients scheduled between 10:30 a.m. and 12:00 p.m. started ingesting at 07:00 a.m. All patients were instructed to consume 500 ml of solution every 30 minutes and drink 500 ml of water after drinking 1 L of solution. Finally, patients took total 2 L of solution and 1 L of water overall for bowel preparation. Patients with afternoon colonoscopy started ingesting the first half of the bowel preparation solution (1 L) at 06:00 a.m. and the second half (1 L) at 10:00 a.m. the day of colonoscopy. They were also instructed to consume the solution and water in the same way as patients with morning colonoscopy.

### Data collection

All patients were asked to answer a questionnaire assessing the factors associated with inadequate bowel preparation, including the amount of solution the patient consumed, the percentage of low-residue diet during the 3 days before colonoscopy, the total number of fecal excretions after the last solution intake, and the color of the last rectal effluent were asked through this questionnaire. Patients who could not drink at least 75% of the solution were regarded as noncompliant and were excluded from the study (n = 3). To assess low-residue diet adherence, patients were asked to report the low-residue diet percentage for the 3 days prior to colonoscopy using a scale from 0% to 100% for their entire diet during this period. The last rectal effluent was characterized as follows: 1, clear liquid; 2, semi-clear liquid; 3, brown liquid; and 4, brown solid^25^. The following clinical data were also collected from each patient: age, sex, body mass index, indication for the procedure, medical history of constipation or laxative use, history of abdominal or gynecologic surgery, previous colonoscopy experience, previous history of inadequate bowel preparation or diverticulosis, other medical comorbidities including diabetes, hypertension, stroke, cirrhosis, and co-medication. Patients privately completed the questionnaire anonymously before colonoscopy and submitted the questionnaires to the hospital staff.

### Assessment of bowel preparation

The colonoscopy procedures were performed by five experienced endoscopists who were blinded to patient information. The endoscopists met several times to ensure uniformity regarding bowel cleansing assessment using recorded videos of sample cases. Immediately after colonoscopy, bowel cleansing was evaluated by the endoscopists using the four-point score scale as follows: 1, poor (large amounts of fecal residue requiring additional cleansing); 2, fair (enough feces or fluid to prevent a completely reliable exam); 3, good (small amount of feces or fluid not interfering with the exam); and 4, excellent (no more than small amounts of adherent feces/fluid). Scores of 3 and 4 were considered as ‘adequate’ and scores of 1 or 2 as ‘inadequate’ preparation.

### Statistical analysis

For categorical variables, a Pearson chi-square test was used. Student t-tests were used to compare the means of continuous variables, and continuous variables are presented as the mean ± standard deviation (SD). Variables with p values lower than 0.1 in univariate analysis were subsequently tested in multivariate logistic regression analysis. For all comparisons, two-sided p-values < 0.05 were considered statistically significant. Statistical analyses were performed using SPSS version 18.0 (SPSS, Chicago, IL).

## Data Availability

No restriction on the availability of materials or information.
